# Sunitinib and Pterostilbene Combination Treatment Exerts Antitumor Effects in Gastric Cancer via Suppression of PDZD8

**DOI:** 10.3390/ijms23074002

**Published:** 2022-04-04

**Authors:** Yudai Hojo, Shingo Kishi, Shiori Mori, Rina Fujiwara-Tani, Takamitsu Sasaki, Kiyomu Fujii, Yukiko Nishiguchi, Chie Nakashima, Yi Luo, Hisashi Shinohara, Hiroki Kuniyasu

**Affiliations:** 1 Department of Molecular Pathology, Nara Medical University, 840 Shijo-cho, Kashihara 634-8521, Nara, Japan; yudaihojo@outlook.com (Y.H.); nmu6429@yahoo.co.jp (S.K.); m.0310.s.h5@gmail.com (S.M.); rina_fuji@naramed-u.ac.jp (R.F.-T.); takamitu@fc4.so-net.ne.jp (T.S.); toto1999-dreamtheater2006-sms@nifty.com (K.F.); yukko10219102@yahoo.co.jp (Y.N.); c-nakashima@naramed-u.ac.jp (C.N.); 2 Department of Surgery, Hyogo College of Medicine, 1-1 Mukogawa-cho, Nishinomiya 663-8501, Hyogo, Japan; 3 Key Laboratory of Neuroregeneration of Jiangsu and Ministry of Education, Co-Innovation Center of Neuroregeneration, Nantong University, Nantong 226001, China; lynantong@hotmail.com

**Keywords:** sunitinib, pterostilbene, PDZD8, mitochondria, oxidative stress

## Abstract

The use of molecular-targeted drugs in the treatment of gastric cancer is increasing. However, the variety of molecular-targeted drugs in gastric cancer is still limited, and the development of new molecular-targeted therapies is required. The effect of combining sunitinib (SUN) with pterostilbene (PTE) on the human gastric cancer cell lines TMK1 and MKN74 was examined in in vitro and in vivo. Compared with SUN or PTE treatment alone, cotreatment induced pronounced suppression of cell proliferation, with a marked increase in oxidative stress. SUN was associated with a significant retention of mitochondrial Fe^2+^. SUN-treated cells decreased expression of PDZ domain-containing protein 8 (PDZD8). Knockdown of PDZD8 in both cells induced Fe^2+^ retention, and siPDZD8+PTE markedly suppressed cell proliferation with suppressed oxidative phosphorylation, as did the combination of SUN+PTE. In a nude mouse tumor model, a pronounced antitumor effect was observed with SUN+PTE treatment compared to SUN alone. PDZD8 may be a newly discovered off-target for SUN, and that the combined use of PTE with SUN significantly promotes antitumor activity in gastric cancer cell lines. The combined use of SUN and PTE might be a new molecular-targeted therapy for gastric cancer.

## 1. Introduction

Gastric cancer is currently the third most common cause of cancer-related death in Japan [1]. The overall 5-year survival rate for this disease has risen to 73.1% due to improvements in early diagnosis and an increase in endoscopic treatments [2,3]. However, advanced gastric cancer (stages III and IV) still has a poor prognosis, with a 5-year survival of 47.2% and 7.3%, respectively [2,3]. Recently, there has been an emphasis on the importance of interdisciplinary treatment with major chemotherapeutic agents such as cisplatin, 5-fluorouracil (5-FU), and taxane [3].

In contrast, the currently recommended molecular-targeted therapies for gastric cancer are trastuzumab, which targets erb-b2 receptor tyrosine kinase 2 (ERRB2) (also known as HER2), and ramucirumab, which targets kinase insert domain receptor (KDR) (also known as VEGFR2) [3]. The survival rate of patients with HER2-positive gastric cancer is 22% and can be prolonged with trastuzumab [4]. In addition, the results of a Phase III clinical trial with ramucirumab showed that it extended median overall survival (OS) [5].

Sunitinib (SUN) is a multitargeted tyrosine kinase inhibitor (TKI). Multitargeted TKIs have been used to treat a variety of malignancies and have shown efficacy [6] in improving the treatment of chronic myelogenous leukemia, gastrointestinal stromal tumors, and renal cancer [7]. SUN inhibits several receptor tyrosine kinases, especially those involved in angiogenesis, such as vascular endothelial growth factor receptor, platelet-derived growth factor receptor, stem cell factor receptor, and FMS-like tyrosine. It has also been shown to inhibit kinase-3, glial cell-line-derived neurotrophic factor receptor, and receptor of macrophage-colony stimulating factor [8,9]. The antitumor activity of SUN has been reported in hepatocellular carcinoma, neuroendocrine gastroenteropancreatic tumors, and non-small-cell lung cancer [9]. However, SUN has not been sufficiently effective against gastric cancer in Phase III clinical trials [10]. To enhance the efficacy of molecularly targeted drugs, they are generally used in combination with other chemotherapeutic agents and other drugs. However, in this case, not only the efficacy but also the synergistic increase in side effects is problematic. We decided to examine the effects of SUN in combination with food nutrients, which have little antitumor effect alone but no side effects, in order to enhance the efficacy of SUN.

PTE is a dietary nutrient that is abundant in blueberries [11]. PTE induces apoptosis by suppressing the proliferation of cancer cells in a concentration-dependent manner, lowering mitochondrial membrane potential, activating cytochrome C, and overinflux of Ca^2+^ [12]. We have also reported that PTE has strong anticancer stem cell activity [13].

In this study, we focused on mitochondrial function and examined alterations in antitumor effect due to the combined use of SUN and PTE.

## 2. Results

### 2.1. Effect of SUN and PTE on Gastric Cancer Cells

**Figure 1:**
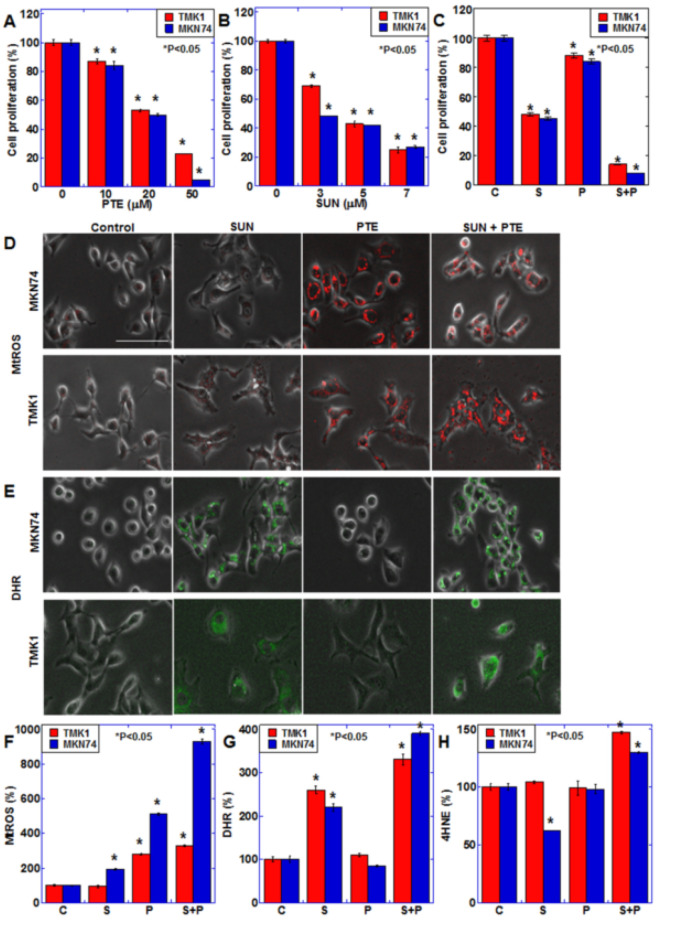
Effect of SUN and PTE cotreatment on cell proliferation in gastric cancer cells. (**A**,**B**) Effect of SUN alone on cell proliferation in TMK1 (**A**) and MKN74 (**B**) cells. (**C**) Effect of cotreatment with SUN (5 μM) and PTE (10 μM) on cell proliferation. (**D**,**E**) Effect of cotreatment with SUN and PTE on mitochondrial superoxide (MtROS, **D**) and H_2_O_2_ (DHR, **E**) levels. (**F**–**H**) Quantification of the fluorescence intensity of MtROS (**F**) and DHE (**G**), and 4-hydroxynonenal (4HNE, H) by ELISA. Error bars represent standard deviations from three independent evaluations. Statistical difference was calculated by ordinary ANOVA. * Statistical difference from control. Scale bar, 50 μm. SUN, sunitinib; PTE, pterostilbene; C, control; S, sunitinib; P, pterostilbene; S+P, cotreatment with SUN and PTE; DHR, dihydrorhodamine 123; MtROS, mitochondrial ROS; HNE, hydroxynonenal.

To examine the effects of SUN and PTE on gastric cancer, the TMK1 and MKN74 human gastric cancer cell lines were treated with various concentrations of each drug for 48 h (Figure 1A,B). Both SUN and PTE inhibited cell proliferation of these cell lines in a dose-dependent manner. Furthermore, cotreatment with SUN and PTE showed a synergistic inhibitory effect compared with the administration of SUN or PTE alone (Figure 1C).

Since PTE induces oxidative stress when administered alone [13], we examined the effect of SUN and/or PTE treatment on oxidative stress (Figure 1D–H). Mitochondrial superoxide (MtROS) levels increased with PTE alone, and exhibited a further increase with PTE and SUN cotreatment. Similarly, mitochondrial hydrogen peroxide (DHR) levels increased with SUN alone and were further increased by the cotreatment. Interestingly, lipid peroxide (4HNE) levels increased only with the cotreatment. These results suggest that the combined use of PTE and SUN induces oxidative stress and promotes cancer cell damage.

**Figure 2:**
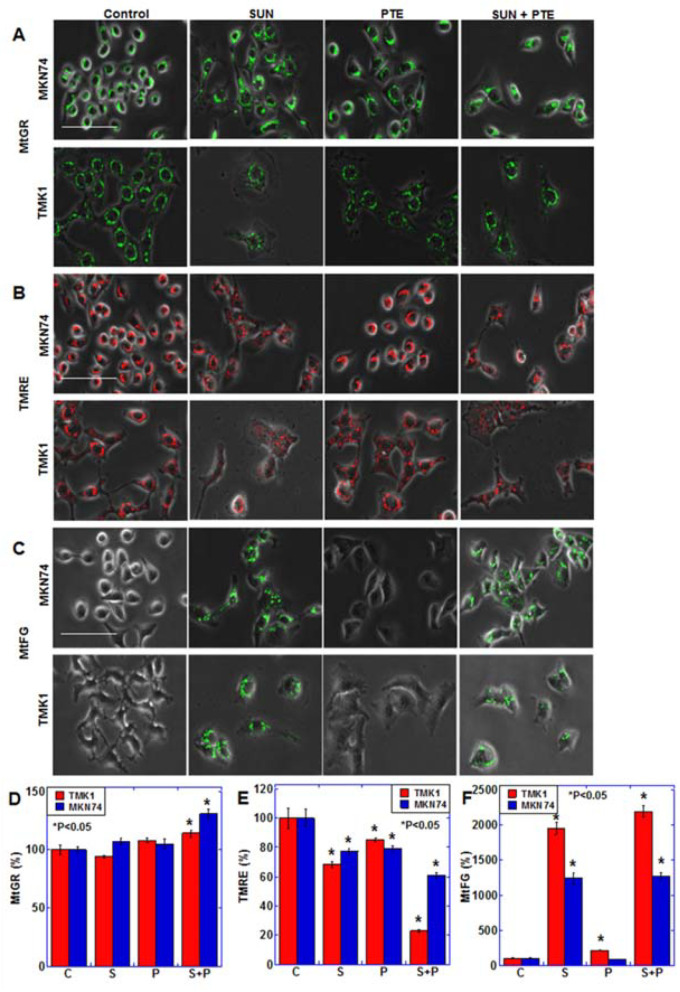
Effect of SUN and PTE cotreatment on mitochondrial function in gastric cancer cells. (**A**–**C**) Effect of cotreatment with SUN (5 μM) and PTE (10 μM) on mitochondrial volume (MtGR, A), mitochondrial membrane potential (TMRE, B) and mitochondrial Fe^2+^ (MtFG, **C**). (**D**–**F**) Quantification of the fluorescence intensity of MtGR (**D**), TMRE (**E**) and MtFG (**F**). Error bars represent standard deviations from three independent evaluations. Statistical difference was calculated by ordinary ANOVA. * Statistical difference from control. Scale bar, 50 μm. SUN, sunitinib; PTE, pterostilbene; C, control; S, sunitinib; P, pterostilbene; S+P, cotreatment with SUN and PTE; MtGR, mitoGreen; TMRE, tetrathylrhodamine ethyl ester; MtFG, mitoFerrogreen.

We therefore next investigated the effect of PTE and SUN cotreatment on mitochondrial function (Figure 2). Mitochondrial mass (MtGR) was not altered with PTE or SUN treatment alone; however, it increased slightly with PTE + SUN cotreatment (Figure 2A,D). The mitochondrial membrane potential (TMRE) decreased slightly with PTE or SUN alone, whereas it decreased markedly in response to cotreatment (Figure 2B,E). Furthermore, when mitochondrial Fe^2+^ (MtFG) was examined, a marked increase was observed with SUN alone, as well as with SUN and PTE cotreatment (Figure 2C,F).

### 2.2. Effect of SUN on Mitochondrial Function

Since SUN treatment induced significant changes in mitochondrial function, we screened TMK1 and MKN74 cells treated with SUN for alterations in the gene expression profile (Table 1). Transcriptome analysis revealed altered expression of metabolism-related genes, indicating that glycolysis and glutaminolysis were enhanced, along with increased conversion of pyruvic acid to acetyl-CoA, which suggest a reprogramming of energy metabolism to lactate fermentation. We also observed changes in the expression of metal ion metabolism-related genes, in line with the effect of SUN treatment in stimulating mitochondrial Fe^2+^ accumulation (Table 2). The list of differentially expressed genes included the mitochondria-related genes, translocase of inner mitochondrial membrane 10B (*TIMM10B*) and *PDZD8*. We further narrowed our focus to genes related to mitochondria and metal ion metabolism from the group of genes whose expression is altered by SUN treatment, and two genes, TIMM10B and PDZD8, were extracted. We compared the expression of the two genes and found that the expression of PDZD8 was about 8-fold higher than that of TIMM10B (Figure S1A), so we examined the expression of PDZD8.

**Figure 3:**
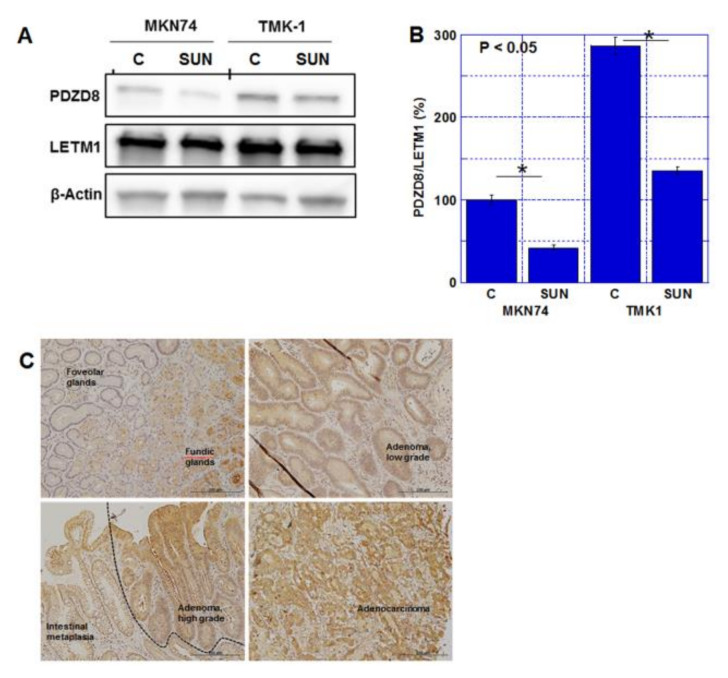
Expression of PDZD8 in gastric cancer cell lines and human gastric cancer tissues. (**A**) Protein levels of PDZD8 in the mitochondrial fraction of TMK1 and MKN74 cells. LETM1 expression was used as a marker of mitochondrial protein purification and a loading control. (**B**) Semi-quantification of the signal densities in the Western blot (panel A). PDZD8 was standardized by β-Actin and LetM1. Error bars represent standard deviations from three independent evaluations. Statistical difference was calculated by ordinary ANOVA. (**C**) Protein levels of PDZD8 in human gastric tissues, foveolar glands and fundic glands (left upper panel), low-grade adenoma (right upper panel), high-grade adenoma and intestinal metaplasia (left lower panel), and adenocarcinoma (moderately differentiated type, right lower panel). Scale bar, 200 μm. PDZD8, PDZ domain-containing protein 8; LETM1, leucine zipper and EF-hand containing transmembrane protein 1; C, control; SUN, sunitinib. * *p* < 0.05.

SUN treatment resulted in a decrease in PDZD8 protein levels in both gastric cancer cell lines in whole-cell lysate (Figure 3A) and mitochondrial fraction (Figure S1B). PDZD8 was physically associated with mitochondrial voltage-dependent anion channel 1 (VDAC1) (Figure S1C). The expression of PDZD8 was detected in adenocarcinomas but not in normal gastric mucosa, intestinal metaplasia, and adenomas (Figure 3B, Table 3). PDZD8 expression in gastric cancer was correlated with invasion of primary tumors (T factor) and pathological stage. However, no correlation was found between nodal metastasis (N factors) and histological grades. The expression level of PDZD8 was low in normal gastric mucosa, intestinal metaplasia, and adenoma, but was present at high levels in adenocarcinomas (Figure 3C, Table 4).

### 2.3. Role of PDZD8 in Gastric Cancer Cells

**Figure 4:**
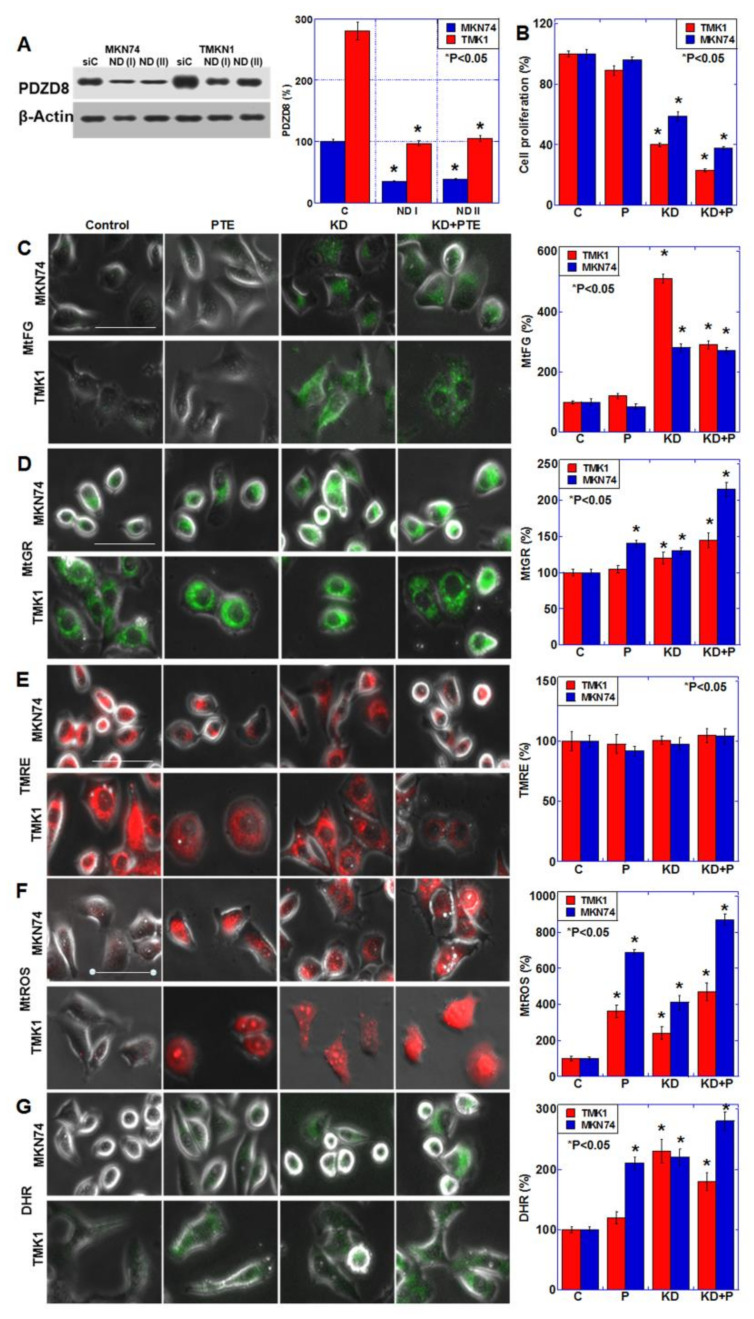
Effect of PDZD8 knockdown on mitochondrial function in gastric cancer cells. (**A**) Effect of *PDZD8* knockdown on protein levels of PDZD8. Right panel, semi-quantification of the Western blot. (**B**) Effect of PDZD8 knockdown plus PTE treatment on cell proliferation. (**C**–**G**) Effect of PDZD8 knockdown plus PTE treatment on mitochondrial Fe^2+^ (MtFG, **C**), mitochondrial volume (MtGR, D), mitochondrial membrane potential (TMRE, **E**), mitochondrial superoxide (MtROS, **F**) and H_2_O_2_ (DHR, **G**) levels. Bar graphs show quantifications of the fluorescence intensities. Error bars represent standard deviations from three independent evaluations. Statistical difference was calculated by ordinary ANOVA. * Statistical difference from control. Scale bar, 20 μm. PDZD8, PDZ domain-containing protein 8; siRNA, small interfering RNA; C, control; PTE, pterostilbene; P, pterostilbene; KD, knockdown of PDZD8; KD+P, cotreatment with PDZD8 knockdown and pterostilbene; KD+PTE, cotreatment with PDZD8 knockdown and pterostilbene; MtFG, mitoFerrogreen; MtGR, mitoGreen; TMRE, tetrathylrhodamine ethyl ester; MtROS, mitochondrial ROS; DHR, dihydrorhodamine 123.

PDZD8 is considered to be a MAM protein, but its role in cancer cells has not been clarified thus far. Therefore, we knocked down *PDZD8* using siRNA and examined its role in the gastric cancer cell lines (Figure 4). Figure 4A demonstrates successful knockdown of PDZD8 protein levels. Mitochondrial PDZD8 also decreased by *PDZD8* knockdown (Figure S1D). *PDZD8* knockdown decreased cell proliferation in both cell lines, and there was a synergistic decrease in proliferation when knockdown cells were also treated with PTE (Figure 4B). *PDZD8* knockdown induced a retention in mitochondrial Fe^2+^, similar to the characteristic retention of mitochondrial Fe^2+^ associated with SUN treatment (Figure 4C). We also examined the *PDZD8* knockdown cells for changes in mitochondria and oxidative stress (Figure 4D–G). The mitochondrial volume increased significantly in response to *PDZD8* knockdown and PTE treatment (Figure 4D). However, the mitochondrial membrane potential was not significantly altered by *PDZD8* knockdown or PTE (Figure 4E). Mitochondrial superoxide increased in the *PDZD8* knockdown cells but it was substantially higher in PTE-treated cells, and there was a marked increase in knockdown cells treated with PTE (Figure 4F). In contrast, mitochondrial hydrogen peroxide levels showed a more pronounced increase in *PDZD8* knockdown cells than in PTE-treated cells, and a marked increase was observed when *PDZD8* knockdown was combined with PTE treatment (Figure 4G).

### 2.4. Effects of SUN Treatment or PDZD8 Knockdown on Mitochondrial Respiration

**Figure 5:**
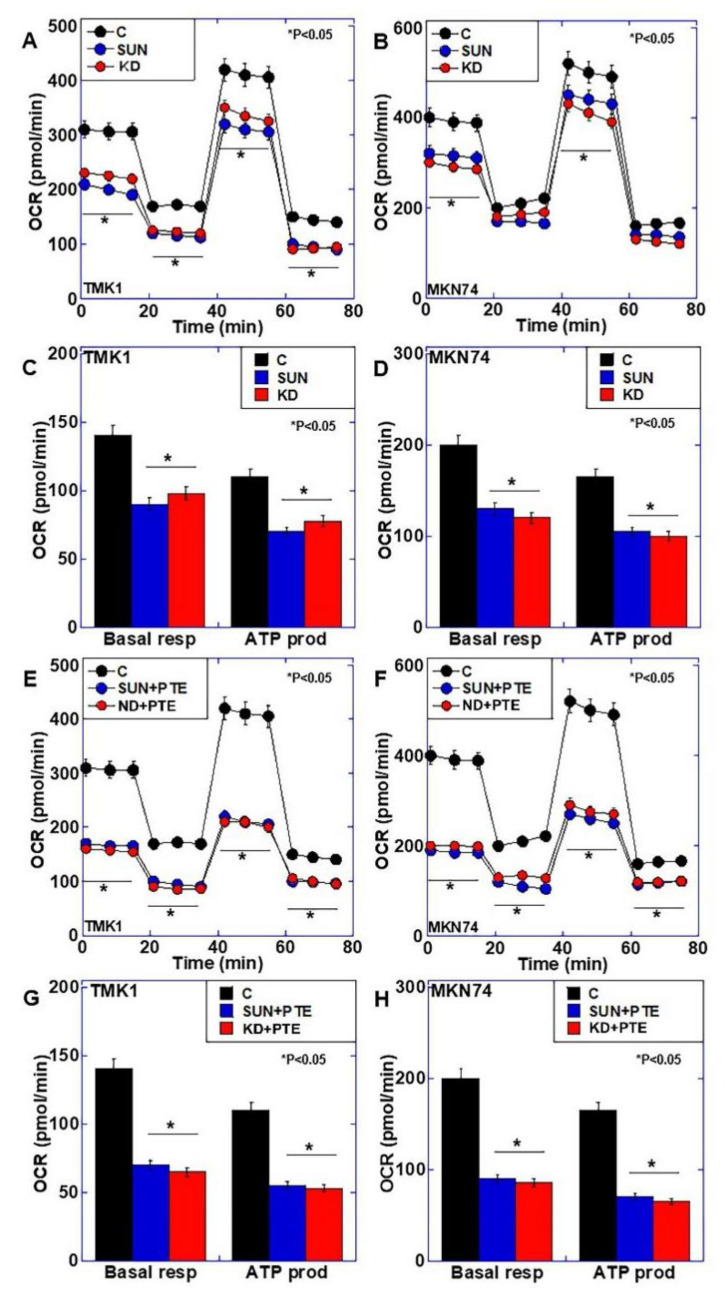
Effect of SUN, PDZD8 knockdown, and cotreatment with PTE on mitochondrial energy metabolism in gastric cancer cells. (**A**,**B**) Flux analysis of SUN-treated or siPDZD8 TMK1 (**A**) and MKN74 (**B**) cells. (**C**,**D**) Basal respiration (Basal resp) and ATP production (ATP prod) of SUN-treated or siPDZD8 TMK1 (**C**) and MKN74 (**D**) cells. (**E**,**F**) Flux analysis of SUN+PTE-treated or siPDZD8 + PTE-treated TMK1 (**E**) and MKN74 (**F**) cells. (**G**,**H**) Basal respiration and ATP production of SUN+PTE-treated or siPDZD8 + PTE-treated TMK1 (**G**) and MKN74 (**H**) cells. Error bars represent standard deviations from three independent evaluations. Statistical difference was calculated by ordinary ANOVA. * Statistical difference from control. SUN, sunitinib; PTE, pterostilbene; PDZD8, PDZ domain-containing protein 8; OCR, oxygen consumption rate; KD, knockdown of PDZD8.

SUN treatment and *PDZD8* knockdown both resulted in mitochondrial iron retention and increased oxidative stress, and we therefore examined their effects on mitochondrial respiration (Figure 5A–D). Both SUN treatment and *PDZD8* knockdown reduced oxidative phosphorylation, and both basal respiration and ATP production were reduced to approximately 70% of the control in both cancer cell lines. Next, we examined the effect of PTE combined with SUN treatment or *PDZD8* knockdown on mitochondrial respiration (Figure 5E–H). Significant suppression of oxidative phosphorylation was observed with SUN+PTE treatment and *PDZD8* knockdown + PTE treatment compared with SUN or siPDZD8 alone. Both basal respiration and ATP production were reduced to approximately 40% of the control in both cell lines treated with SUN+PTE or *PDZD8* knockdown + PTE. These results suggest that SUN suppresses PDZD8 protein levels, as well as mitochondrial respiration via mitochondrial Fe^2+^ retention, and induces an increase in oxidative stress. PTE is thought to synergistically facilitate these processes when combined with SUN treatment.

### 2.5. Effect of Extrinsic PDZD Protein on SUN and PTE-Induced Cell Growth Inhibition in TMK1 Cells

**Figure 6:**
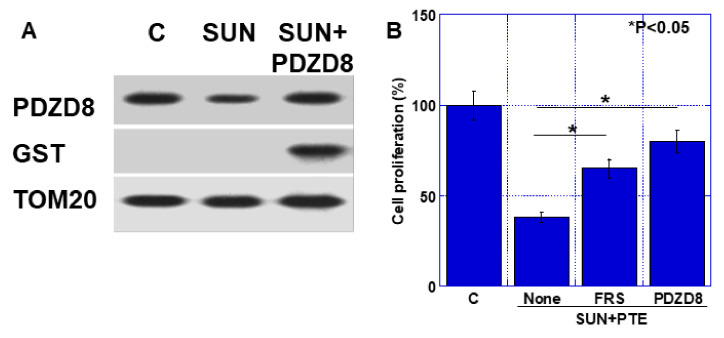
Effect of extrinsic PDZD protein on SUN and PTE-induced cell growth inhibition. (**A**) GST-tagged human recombinant PDZD8 protein was administrated by Chariot^TM^ protein delivery reagent. PDZD8 protein level was examined by Western blotting using mitochondrial fraction of TMK1 cells. TOM20 was subjected as a mitochondrial marker. TMK1 cells were treated with SUN and PTE with ferrostatin (FRS) or hrPDZD8. (**B**) Semi-quantification of panel A. Error bars represent standard deviations from three independent evaluations. Statistical difference was calculated by ordinary ANOVA. SUN, sunitinib; PTE, pterostilbene; PDZD8, PDZ domain-containing protein 8.

By extrinsic administration of GST-tagged human recombinant PDZD8 (hrPDZD), decreased mitochondrial PDZD8 protein by SUN was recovered to the control level (Figure 6A). Extrinsic hrPDZD8 was confirmed by detection of GST. As shown in Figure 6B, cell growth inhibition by SUN+PTE was recovered by ferrostatin (FRS, 44% recover) and by extrinsic hrPDZD8 (68%).

### 2.6. Effect of SUN and PTE on Mouse Tumors

**Figure 7:**
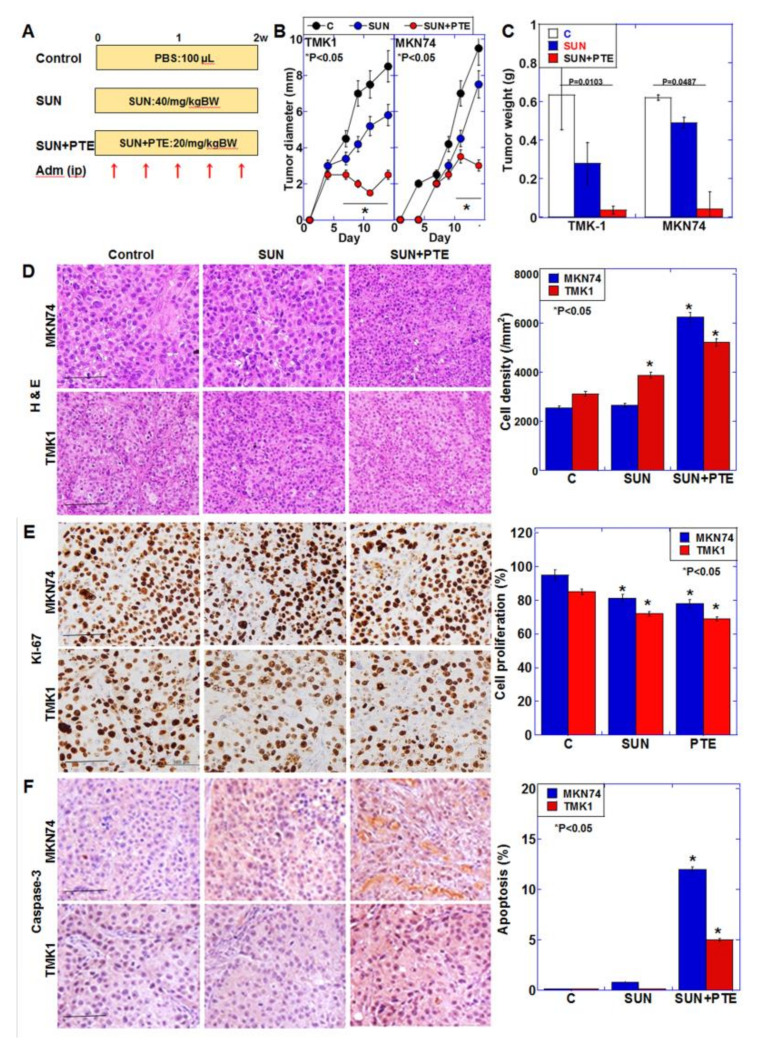
Effect of SUN and PTE cotreatment on tumor growth of gastric cancer cell lines in nude mice. (**A**) Protocol of the animal experiment. Nude mice (3 per group) were treated with SUN (40/mg/kg BW, ip) and/or PTE (20/mg/kg BW, ip), 5 times over 2 weeks. (**B**) Tumor diameter over time. (**C**) Tumor weight at euthanasia, 2 weeks after inoculation. (**D**–**F**) Examination of tumors: histology by H & E staining (**D**), cell proliferation by Ki-67 immunohistochemistry (**E**) and apoptosis by cleaved caspase-3 immunohistochemistry (**F**). Bar graphs show quantification of the cell density (**D**) and positive cell frequency (**E**,**F**). Error bars represent standard deviations from three independent evaluations. Statistical difference was calculated by ordinary ANOVA. * Statistical difference from control. Scale bar, 100 μm. SUN, sunitinib; PTE, pterostilbene; SUN+PTE, cotreatment with SUN and PTE; BW, body weight; ip, intraperitoneal injection; H & E, hematoxylin and eosin.

In order to examine the antitumor effect of SUN+PTE, TMK1 and MKN74 cells were subcutaneously inoculated into nude mice, and SUN alone or SUN+PTE were administered for 2 weeks (Figure 7A). Figure 7B,C shows that SUN alone exerted a tumor growth inhibitory effect of 20% and 60% on TMK1- and MKN74-derived tumors, respectively. In contrast, the combined administration of SUN and PTE showed a 95% tumor growth inhibitory effect on tumors from both cell types. The combined administration of SUN and PTE shrank tumor cells, increased cell density (Figure 7D), decreased proliferative capacity (Figure 7E), and increased apoptosis (Figure 7F). When the weights of various organs from cancer-bearing mice were measured, the weights of the liver and QCM were significantly reduced by treatment with SUN alone, suggesting tissue damage (Figure S2). In contrast, when PTE was used in combination with SUN, the weight of these organs was restored to the level of the control. This suggests that PTE promotes the antitumor effect of SUN, but has a protective effect on normal tissues and may reduce the toxicity of SUN.

## 3. Discussion

SUN is a multitargeted TKI, with a mechanism of action that is common to almost all TKIs, in that it binds to the catalytic binding site of multiple tyrosine kinases and competes with ATP [6]. In contrast to other TKIs, SUN is known to cause mitochondrial damage, including mitochondrial respiratory chain uncoupling, and alterations in oxidative phosphorylation, the Krebs cycle, mitochondrial DNA replication, and ADP/ATP translocation [14]. Such mitochondrial damage ultimately lowers the mitochondrial membrane potential, resulting in decreased energy production and increased oxidative stress, which leads to cell death [14,15]. However, it was not clear whether such mitochondrial disorders were associated with the antitumor effects of SUN. In our experiments, SUN alone does not exert as much mitochondrial damage as the combination of SUN+PTE treatment; the combined use of SUN and PTE resulted in the retention of Fe^2+^ in mitochondria and the induction of high ROS production, leading to cell death.

In our study, SUN and *PDZD8* knockdown both induced mitochondrial Fe^2+^ retention. PDZD8 is a protein in the mitochondria-associated endoplasmic reticulum (ER) membrane (MAM). MAM is the region of the ER which adjoins the mitochondrial membrane. This special ER domain is closely related to the outer membrane of the mitochondrion due to protein tethering, which enables the construction of signaling platforms that generate or regulate various cellular functions such as lipid biosynthesis, Ca^2+^ homeostasis, inflammation, autophagy, and apoptosis [16,17]. Importantly, in pathological conditions such as cancer, neurodegenerative diseases, and metabolic syndrome, changes in MAM composition and plasticity have been shown to alter the function of ER-mitochondrial crosstalk [16,18]. PDZD8 is a synaptotagmin-like mitochondrial lipid binding domain protein that is involved in the tethering of these membrane contact sites through interaction with other proteins and membrane lipids [19]. Recent studies have suggested that PDZD8-associated MAM is involved in the transport of specialized lipids and Ca^2+^, and is involved in neurite outgrowth and the regulation of dendrite Ca^2+^ dynamics [20,21]. Moreover, the MAM is also required for iron homeostasis. When this membrane component is lost, the Aft1-dependent iron deficiency reaction is activated even in an iron-rich state, and excess iron accumulates in the cells [22]. This function is not related to the known role of MAM in calcium regulation, phospholipid biosynthesis, or its effect on mitochondrial morphology [22]. From these findings, we hypothesize that the SUN-induced accumulation of Fe^2+^ in mitochondria observed in this study may have been due to disruption of PDZD8.

The decrease in PDZD8 mRNA and protein expression by SUN is an interesting result, but the cause of this decrease could not be determined in this study. In guessing the cause, the discrepancy between PDZD8 mRNA expression and PDZD8 as a MAM protein is thought to be the key. That is, in the present study, PDZD8 protein was expressed specifically in gastric cancer, but not in normal gastric tissue. However, previous mRNA expression profiling data in gastric cancer have not shown that PDZD8 is highly expressed in gastric cancer. This dissociation between mRNA and protein expression levels predicts that PDZD8 is recruited to MAM, which stabilizes the protein. In contrast, MAM proteins other than PDZD8, such as mitofusin-2, probably play a role in normal tissues.

Mitochondrial NEET, another protein localized to the MAM, is involved in the regulation of multiple processes such as autophagy, apoptosis, ferroptosis, oxidative stress, cell proliferation, redox regulation, and especially iron and iron-sulfur homeostasis [23]. We found that VDAC1 co-precipitated with PDZD8 in immunoprecipitation analyses. It has been reported that mtNEET docks with VDAC1 to regulate gating of ion channels [24], which suggests that MAM-associated PDZD8 might be involved in and regulate mitochondrial iron metabolism via mitochondrial NEET. The impairment of PDZD8 may thus reduce mitochondrial NEET function and result in mitochondrial iron accumulation. Furthermore, the results from our study show that PDZD8 protein is specifically expressed in gastric cancer, and no increase in PDZD8 protein was observed in non-cancerous tissues such as adenomas or intestinal metaplasia. This indicates that MAM formation by PDZD8 might be characteristic of undifferentiated and proliferative tissues such as cancer. Indeed, mitochondrial NEET plays a critical role in promoting cancer proliferation and metastasis [25].

PTE is a natural dimethylated analog of resveratrol [11] and is said to have a stronger antioxidant effect in differentiated cells [11,26]. In contrast, PTE strongly induces ROS production in cancer cells even at low concentrations [13]. PTE has been reported to decrease mitochondrial membrane potential, increase oxidative stress, and alter mitochondrial calcium ion levels in cancer cells, leading to the induction of apoptosis [27,28,29]. In contrast, in ferroptosis, the inducer elastin opens VDAC, induces mitochondrial hyperpolarization and ROS production, and induces cell death [30,31]. Our previous study suggested that PTE induces ROS production due to hyperpolarization of mitochondrial membrane potential, resulting in a mixture of ferroptosis and apoptosis [13]. Although ferroptosis is induced by lipid peroxidation [32], cell death by PTE can be rescued by suppressing the production of lipid peroxide during PTE treatment [13]. Interestingly, in our study, we observed Fe^2+^ accumulation in mitochondria when PDZD8 was suppressed by SUN treatment or knockdown. Fe^2+^ generates hydroxyl radicals via the Fenton reaction and causes the accumulation of lipid peroxide [33], which is considered to be a background factor for inducing ferroptosis [34]. Furthermore, our data showed that ferrostatin, a ferroptosis inhibitor, recovered the inhibition of cell proliferation by SUN+PTE. These findings suggest that the combined use of SUN and PTE promotes suppression of PDZD8 by SUN, resulting in iron retention in mitochondria and promoting the ferroptosis-inducing effect of PTE.

Toxic effects on organs due to mitochondrial disruption, such as myocardial disorders, have been reported as side effects for SUN, trastuzumab, imatinib, bevacizumab, and sorafenib [14]. Such mitochondrial damage ultimately lowers the mitochondrial membrane potential, resulting in decreased energy production and increased oxidative stress resulting in cell death [14,15]. However, few reports have clarified whether such mitochondrial disorders are directly related to the antitumor effects of these drugs. Furthermore, the identification of a drug that reduces SUN-associated organ damage is a desirable outcome. Our data show that the SUN-induced weight loss of liver and skeletal muscle is suppressed when it is combined with PTE. Longer-term observation of the myocardium in response to SUN+PTE may be necessary. However, our data suggest that the combined use of PTE with SUN not only promotes the antitumor effect of SUN, but also enhances the safety of SUN in normal tissues.

## 4. Materials and Methods

### 4.1. Cell Lines and Reagents

The human gastric carcinoma cell lines TMK1 and MKN74 were a gift from Professor Wataru Yasui (Molecular Pathology, Hiroshima University, Hiroshima, Japan). The human monocytic cell line U937 was purchased from Dainihon Pharmacy Co. (Tokyo, Japan). TMK1 and MKN74 cells were cultured in Dulbecco’s modified Eagle medium (DMEM) (Wako Pure Chemical Corporation, Osaka, Japan) supplemented with 10% fetal bovine serum (FBS) (Sigma-Aldrich Chemical Co., St. Louis, MO, USA) at 37 °C in 5% CO_2_.

Sunitinib (SUN) and pterostilbene (PTE) were purchased from MedChemExpress (Monmouth Junction, NJ, USA) and Tokyo Chemical Industry Co., Ltd. (Tokyo, Japan), respectively. Cells were treated with SUN (5 µM) and/or PTE (10 µM) for 48 h. Ferrostatin-1 (FRS, 2 µM, Funakoshi, Tokyo, Japan) was used for inhibition of ferroptosis.

### 4.2. Cell Growth and Apoptosis

Cell growth was assessed using the 3-(4,5-dimethylthiazol-2-yl)-5-(3-carboxymethoxyphenyl) -2-(4-sulfophenyl)-2H-tetrazolium (MTS)-based Celltiter 96 Aqueous One Solution Cell Proliferation Assay kit (Promega Corporation, Madison, WI, USA), as previously described [13]. The absorbance was measured at 490 nm on a Multiskan FC Microplate Photometer (Thermo Fisher Scientific, Waltham, MA, USA).

### 4.3. Mitochondrial Imaging

Mitochondrial functions were examined using fluorescent probes. Cells were incubated with the probes for 30 min at 37 °C and then imaged using a BZ-X710 All-in-One fluorescence microscope (KEYENCE, Osaka, Japan). We used MitoROS (mtROS) (10 μM, AAT Bioquest Inc., Sunnyvale, CA, USA) and dihydrorhodamine 123 (DHR) (10 μM, Sigma-Aldrich) to assess oxidative stress, mitoGreen (mtGR) (100 nM, PromoCell GmbH, Heidelberg, Germany) to assess mitochondrial volume, tetrathylrhodamine ethyl ester (TMRE) (200 nM, Sigma-Aldrich) to assess mitochondrial membrane potential, and mitoFerrogreen (mtFG) (20 nM, Dojindo, Kumamoto, Japan) to assess mitochondrial iron (Fe^2+^).

### 4.4. mRNA Profiling

TMK1 and MKN74 cells were treated with 5 µM SUN for 48 h. Total RNA was extracted using TRI Reagent (Molecular Research Center, Inc., Cincinnati, OH, USA), according to the manufacturer’s instructions. For mRNA profiling, the RNA was sent to a DNA array service (Filgen, Inc., Nagoya, Japan). For quality control of mRNA, mRNA templates were confirmed that the OD280/260 ratio was more than 1.8, OD230 was more than 1.6 and the S28/S18 ratio was more than 2.0. The data were analyzed using the Microarray Data Analysis Tool Ver3.2 (Filgen).

### 4.5. Extracellular Flux Analysis

To analyze mitochondrial respiration and ATP production, we used a Seahorse XF Analyzer (Agilent Technologies, Santa Clara, CA, USA), which measures extracellular flux in live cells. The cells were collected immediately after treatment, transferred into the wells of an XF plate at densities of 2 × 10^4^ cells/well, and incubated overnight. The following day, the medium in the XF plate was replaced with XF DMEM medium 1 h prior to the assay, and a Mito Stress Test (Seahorse XF Cell Mito Stress Test, Agilent) was performed according to the manufacturer’s protocol. The oxygen consumption rate (OCR) was measured under the following conditions: 2 µM (MKN74) or 3 µM (TMK1) oligomycin, 0.5 µM carbonyl cyanide-p-trifluoromethoxyphenylhydrazone, and 0.5 µM rotenone/antimycin A. The OCR was normalized to the total cellular protein concentration, which was determined after protein extraction from the analyzed cells.

### 4.6. Protein Extraction

To prepare whole-cell lysates, cells were washed twice with cold PBS, harvested, and lysed with RIPA buffer containing 0.1% sodium dodecyl sulfate (SDS) (Thermo Fisher) [35]. Cell fractions were extracted by processing the cells with a Cell Fractionation Kit (Abcam, Cambridge, UK), according to the manufacturer’s instructions [36]. Protein assays were performed using a Protein Assay Rapid Kit (Wako).

### 4.7. Immunoblot Analysis

Whole-cell lysates were prepared as previously described [35]. Lysates (50 μg) were subjected to immunoblot analysis using 12.5% SDS-polyacrylamide gels, followed by electrotransfer onto nitrocellulose membranes (Bio-Rad, Hercules, CA, USA). The membranes were incubated with primary antibodies and then with peroxidase-conjugated IgG secondary antibodies (MBL, Nagoya, Japan). PDZD8 expression was assessed using an antibody against PDZD8 (Bioss Inc., Woburn, MA, USA). Antibodies against β-actin (Oncogene Research Products, Cambridge, MA, USA), leucine zipper and EF-hand containing transmembrane protein 1 (LETM1), and translocase of the outer membrane-20 (TOM20) (Proteintech Group, Inc., Rosemont, USA) were used to assess protein loading. Binding of the immune complex was visualized using a CSA system (DAKO, Carpinteria, CA, USA).

### 4.8. Immunoprecipitation

Immunoprecipitation was performed according to a previously described method [37]. Lysates were pre-cleaned in lysis buffer with protein A/G agarose (Santa Cruz Biotechnology) for 1 h at 4 °C and subsequently centrifuged. The supernatants were then incubated with a precipitation antibody against VDAC1 (Santa Cruz) and protein A/G agarose for 1.5 h at 4 °C. Precipitates were collected by centrifugation, washed thrice with wash buffer, and solubilized with 4× Laemmli Sample Buffer (Bio-Rad, Hercules, CA, USA) and 2-mercaptoethanol (Sigma). Finally, immunoblotting was performed using antibody against PDZD8 (Bioss Inc). For secondary antibody, VerBlot for IP Detection Reagent HRP (Abcam) was used.

### 4.9. Tissue Microarray and Surgical Specimens

A human gastric adenocarcinoma tissue microarray (product ID: BS01012d), which contains 71 cases of gastric adenocarcinoma, was purchased from US Biomax, Inc. (Rockville, MD, USA). We also examined deidentified surgical specimen slides, comprising 14 cases of gastric adenocarcinoma and 11 cases of gastric adenoma (pathologically diagnosed in the Department of Molecular Pathology, Nara Medical University in 2018). Of these cases, 12 contained some areas of metaplastic epithelium. All procedures were performed in accordance with the Ethical Guidelines for Human Genome/Gene Research issued by the Japanese Government and were approved by the Ethics Committee of Nara Medical University (Approval Number 937, 2018/4/1).

### 4.10. Small Interfering RNA

Stealth Select RNAi (siRNA) targeting human *PDZD8* was purchased from Sigma. AllStars Negative Control siRNA was used as a control (Qiagen; Valencia, CA, USA). The cells were transfected with 10 nM siRNA using Lipofectamine 3000 (Thermo Fisher) according to the manufacturer’s recommendations.

### 4.11. Enzyme-Linked Immunosorbent Assay (ELISA)

An ELISA kit (CUSABIO TECHNOLOGY LLC, Houston, TX, USA) was used to measure the concentration of 4-hydroxynonenal (4-HNE). The assay was performed according to the manufacturer’s instructions, using whole-cell lysates.

### 4.12. Extrinsic PDZD8 Protein Administration

GST-tagged human recombinant PDZD8 protein (10 μg, Novus Biologicals, Centennial, CO, USA) was transported to TMK1 cells (1 × 10^6^) by Chariot (Active Motif, Carlsbad, CA, USA) according to the manufacturer’s instructions. After 6 h culture, mitochondrial fraction was extracted from TMK1 cells to detect PDZD8 protein and GST by Western blot using antibodies against PDZD8 (Bioss) and GST (Abcam, EPR4236).

### 4.13. RT-PCR

To assess mRNA expression, RT-PCR was performed with 2 µg total RNA extracted from MKN74 and TMK1 cells using TRI REAGENT (Molecular Research Center, Inc., Cincinnati, OH, USA) according to the manufacturer’s protocol. cDNA was synthesized with 0.5 µg total RNA using a High-Capacity cDNA Reverse Transcription Kit (Applied Biosystems, Waltham, MA, USA). The primer sets for human PDZD8 amplification, TIMM10B and TOM20 were as follows: PDZD8, forward, 5′-TCC TCG TGT TGA TGC TGA AG-3′ and reverse, 5′-TTG TCT GAC GTG TTG GGT GT-3′ (NCBI Reference Sequence: NM_173791.4); TIMM10B, forward, 5′-CAG CAG CAG CAG CAA CAG-3′ and reverse, 5′-AGC TGC CTG ATG GAG AGA CC-3′ (NCBI Reference Sequence: NM_012192.4); TOM20, forward, 5′- ATG GTG GGT CGG AAC AGC-3′ and reverse, 5′- TCT TCA GCC AAG CTC TGA GC-3′ (NCBI Reference Sequence: NM_014765.3); synthesized by Sigma Genosys (Ishikari, Japan). PCR products were electrophoresed on a 2% agarose gel and stained with ethidium bromide. β-Actin mRNA was also amplified for use as an internal control.

### 4.14. Animals

Four-week-old BALB/c Slc-nu/nu mice were purchased from SLC Japan, Inc. (Shizuoka, Japan), and maintained in accordance with both the institutional guidelines approved by the Committee for Animal Experimentation of Nara Medical University, and the current regulations and standards of the Ministry of Health, Labor, and Welfare of Japan (Approval number 12777, 2019/4/6).

### 4.15. Animal Tumor Models

Subcutaneous tumor models were established by inoculating cancer cells (MKN74: 5 × 10^6^ per mouse, TMK-1: 1 × 10^7^ per mouse) into the scapular subcutaneous tissues of nude mice on Day 0. Eighteen mice were randomly divided into 6 groups: Control, SUN alone, and SUN+PTE, for each cell line. SUN (40 mg/kg body weight, diluted with 5% dimethyl sulfoxide) and PTE (20 mg/kg body weight, diluted with 5% dimethyl sulfoxide) were intraperitoneally administered on Days 4, 7, 9, 11, and 13. Mice in the control group were injected with 100 µL of phosphate-buffered saline (PBS; Wako) into the intraperitoneal cavity. Tumor diameter was measured over the skin of each mouse on each treatment day. The mice were sacrificed on Day 16, and the organs and quadriceps femoris muscle (QFM) were immediately excised and weighed.

### 4.16. Immunohistochemistry

Tumors excised at 4 h after treatment from mice belonging to the different groups were fixed in 10% paraformaldehyde for 24 h and paraffin-embedded. Consecutive sections were processed for immunohistochemical analysis of PDZD8 (Bioss), Ki-67 (Abcam, ab16667), and cleaved caspase-3 (Cell Signaling Technology, #9661). For evaluation of PDZD8, we counted cells that exhibited immunoreactivity in the cytoplasm, and scored staining strength as negative, low, moderate, or high. For evaluation of Ki-67 and caspase-3 immunoreactivity, positive cells were counted in five high-power fields of view, in three sections from each group.

### 4.17. Statistical Analysis

Statistical significance was calculated using a two-tailed Fisher’s exact test or an ordinary analysis of variance (ANOVA), using InStat software (GraphPad, Los Angeles, CA, USA). Correlations were tested using Pearson’s correlation test. A two-sided *p* value of <0.05 was considered to indicate statistical significance.

## 5. Conclusions

**Figure 8:**
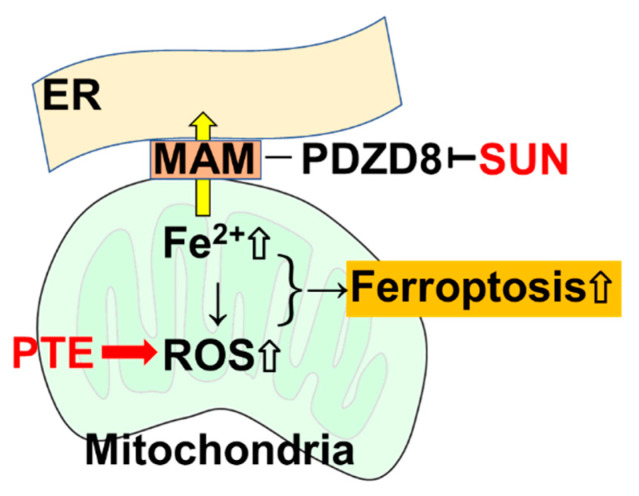
SUN inhibits the MAM protein PDZD8 as an off-target, resulting in inhibition of the translocation of Fe^2+^ into the endoplasmic reticulum in mitochondria, leading to Fe^2+^ retention in mitochondria. PTE increases the production of mitochondrial ROS, but ROS production is further enhanced due to Fe^2+^ accumulated by SUN. As a result, ferroptosis is induced. SUN: sunitinib; PTE, pterostilbene; ER, endoplasmic reticulum; MAM, mitochondria-associated endoplasmic reticulum membrane; PDZD8, PDZ domain-containing protein 8; ROS, reactive oxygen species.

The results from our study reveal that PDZD8, a MAM protein, may be a new molecular target for SUN, and the accumulation of mitochondrial Fe^2+^ via PDZD8 suppression enhances the ferroptosis-promoting effect of PTE, exerting a new antitumor effect (Figure 8). The mechanisms underpinning the inhibition of PDZD8 by SUN, as well as the mechanisms through which PDZD8 controls mitochondrial iron accumulation could not be fully clarified in this study. However, our findings highlight a new therapeutic strategy for SUN and suggest a new molecular target for the development of new TKIs. More detailed studies are expected to open the way to clinical applications in the future.

## Figures and Tables

**Table 1:** Expression of metabolism-associated genes.

Gene Symbol	MKN74	TMK1	Gene Symbol	MKN74	TMK1
*PCNA*	−2.91	−5.16	*GPT*	1.18	1.15
*SLC38A5*	−1.53	−11.7	*LDHD*	1.21	1.11
*TGM2*	−1.38	−58.84	*SLC16A13*	1.24	1.11
*ME2*	−2.57	−2.33	*ACO1*	1.29	1.24
*CKLF*	−1.64	−4.35	*GAD1*	1.12	1.43
*L2HGDH*	−2.59	−1.93	*ME3*	1.88	1.2
*PDHA1*	−2.58	−1.57	*ACO2*	1.18	1.91
*PFKP*	−2.34	−1.78	*SLC16A5*	1.53	1.58
*CKB*	−1.39	−2.81	*SLC38A10*	1.55	1.59
*PDK1*	−1.12	−2.82	*PDK2*	1.72	1.82
*SLC38A4*	−2.18	−1.21	*SLC38A9*	2.19	1.49
*GAD2*	−1.13	−2.11	*SLC38A2*	2.34	1.46
*TGM1*	−1.65	−1.63	*PDP1*	2.88	1.12
*LDHB*	−1.66	−1.26	*GLS*	4.35	1.11
*SLC16A3*	−1.15	−1.66	*ME1*	2.17	1.69
*GPT2*	−1.38	−1.59	*SLC17A5*	1.65	3.09
*GOT2*	−1.56	−1.1	*SLC16A4*	3.63	2.17
*OGFOD1*	−1.18	−1.52	*PDK4*	1.74	5.81
*PKM*	−1.31	−1.5			
*PDHX*	−1.48	−1.21			
*OGDHL*	−1.17	−1.31			
*CKM*	−1.29	−1.15			
*PFKL*	−1.11	−1.22			

**Table 2:** Expression of metal ion-binding protein-associated genes.

Gene Symbol	MKN74	TMK1	Gene Symbol	MKN74	TMK1
*IRF2BPL*	−3.27	−2.66	*ZNF474*	2.13	1.03
*IRF2BP2*	−2.59	−2	*MSS51*	2.07	1.51
*PLEKHM3*	−2.47	−2.77	*ZNF608*	1.91	1.43
*PLEKHM1*	−2.25	−2.33	*ENPP4*	1.86	−1.13
*TIMM10B*	−2.01	−2.05	*SNRNP48*	1.84	1.74
*ZNF428*	−1.98	1.05	*ITGAE*	1.84	1.73
*LENG9*	−1.79	−1.29	*FREM3*	1.65	1.28
*ASPHD2*	−1.79	−1.09	*PDE6A*	1.65	1.16
*AMDHD1*	−1.64	−1.55	*TOPAZ1*	1.61	1.18
*ZNF609*	−1.56	−1.22	*SLC39A5*	1.61	1.18
*PDZD8*	−1.45	−1.38	*SLC39A8*	1.57	1.63
*MOB2*	−1.44	−1.56	*FRAS1*	1.51	−1.27

**Table 3:** Expression of PDZD8 in 85 gastric cancer cases.

Parameter		n	PDZD8 Expression		High	*p* Value ^(1)^
			Low	Moderate		
Total		85	8	39	38	
Age	32–62 yrs ^(4)^	42	4	18	20	NS ^(2)^
	63–90 yrs	43	4	21	18	
Sex	Male	68	5	31	32	NS
	Female	17	3	8	6	
pT ^(3)^	1	10	3	5	2	0.031
	2	25	4	12	9	
	3	44	1	21	22	
	4	6	0	1	5	
pN ^(3)^	0	65	8	31	26	NS
	1–2	20	0	8	12	
pStage ^(3)^	1	34	7	16	11	0.0106
	2	44	1	22	21	
	3–4	7	0	1	6	
Grade ^(3)^	1	14	3	5	6	NS
	2	24	1	11	12	
	3	47	4	23	20	

^(1)^ *p* value was calculated by ordinary ANOVA test. NS, not significant. ^(2)^ NS, not significant. ^(3)^ pT1, tumor confined within the submucosa; pT2, tumor invades the muscularis propria; pT3, tumor invades the subserosa; pT4, tumor invasion is contiguous to or exposed beyond the serosa or tumor invades adjacent structures; pN0, no regional lymph node metastasis; pN1-2, metastasis in 1 or more regional lymph nodes; pStage 1, pT1 and pN0-1, or pT2 and pN0; pStage 2, pT2 and pN1-2, or pT3 and pN0-1; pStage 3, pT2 and pN3, pT3 and pN2-3, or pT4 and pN0-3; pStage 4, all cases with distant metastasis; G1, well differentiated; G2, moderately differentiated; G3, poorly differentiated [3]. ^(4)^ Younger half of the cases; yrs, years old.

**Table 4:** Expression of PDZD8 in gastric tissues.

Tissue	n	PDZD8 Expression		Moderate	High	*p* Value ^(1)^
		Negative	Low			
Foveolar epithelium	35	35	0	0	0	
Pyloric gland	24	24	0	0	0	
Fundic gland	11	11	0	0	0	
Intestinal metaplasia	12	12	0	0	0	
Adenoma	11	11	0	0	0	
Adenocarcinoma	85	0	8	39	38	<0.0001

^(1)^ *p* value was calculated by ordinary ANOVA test. NS, not significant.

## Data Availability

Not applicable.

## Supplementary Materials

The following supporting information can be downloaded at: https://www.mdpi.com/article/10.3390/ijms23074002/s1.

## Abbreviations

SUN	sunitinib
PTE	pterostilbene
TKI	tyrosine kinase inhibitor
ER	endoplasmic reticulum
MAM	Mitochondria-associated endoplasmic reticulum membrane
PDZD8	PDZ domain-containing protein 8
MMP	mitochondrial membrane potential
OCR	oxygen consumption rate
LETM1	leucine zipper and EF-hand containing transmembrane protein 1
QFM	quadriceps femoris muscle
VDAC1	voltage-dependent anion channel 1
