# Supplementary material for: Sunitinib and Pterostilbene Combination Treatment Exerts Antitumor Effects in Gastric Cancer via Suppression of PDZD8
Source: Int J Mol Sci. 2022 Apr 4;23(7):4002. doi: 10.3390/ijms23074002 (PMC8999764; doi:10.3390/ijms23074002)
Supplement: Supplementary file 1 [file ijms-23-04002-s001.zip › ijms-1649259-supplementary.pdf]

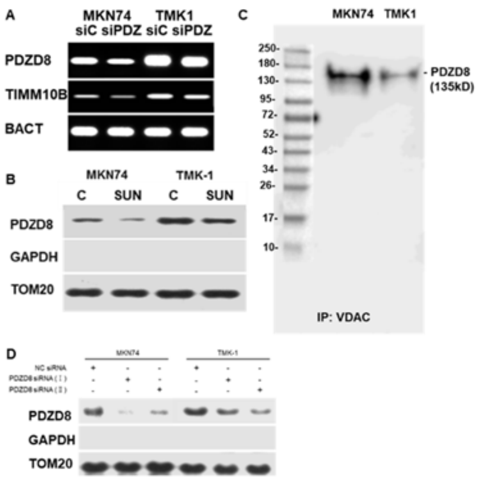

Figure S1. (**A**) Expression of TIMM10B and PDZD8 in TMK1 and MKN74 cells by RT-PCR. (**B**) PDZD8 protein levels in the mitochondrial fraction with (SUN) or without (**C**) sunitinib (SUN) treatment. (**C**) Coprecipitation of PDZD8 with VDAC1 in TMK1 cells. Immunoprecipitant by anti-PDZD8 antibody of TMK1 cell were detected by anti-VDAC1 antibody. (**D**) PDZD8 protein levels in the mitochondrial fraction by treatment with control siRNA or siRNA for PDZD8. GAPDH and TOM20 were subjected as cytosolic and mitochondrial protein marker.

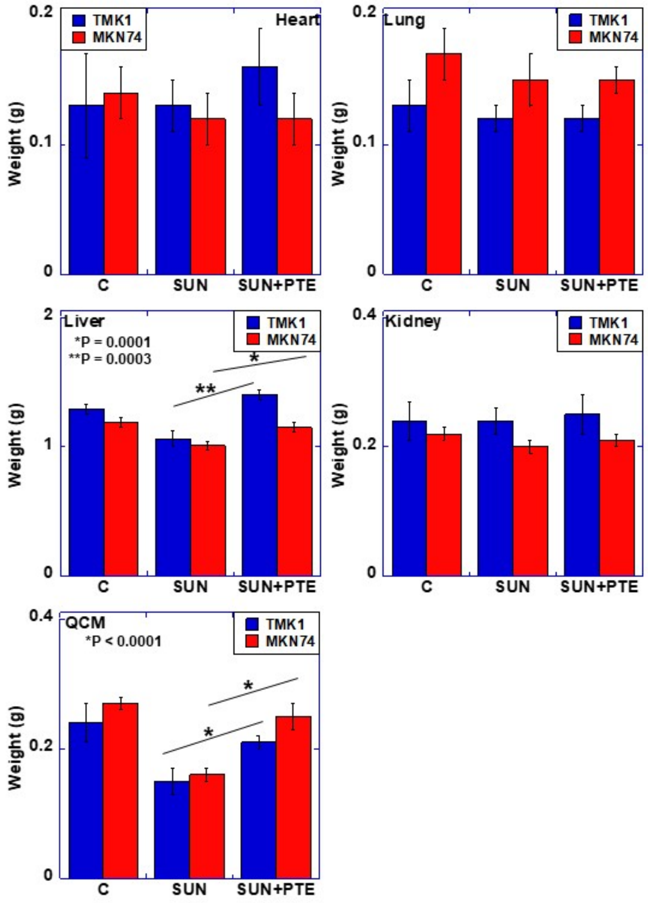

Figure S2. Effect of SUN and PTE cotreatment on organ weight in nude mice. Organ weight of the heart, bilateral lung, liver, bilateral kidneys, and bilateral quadriceps muscle (QCM) in nude mice (3 per group) treated with SUN (40/mg/kg BW, ip), PTE (20/mg/kg BW, ip), or both. Error bars represent

standard deviations from three independent evaluations. Statistical difference was calculated by ordinary ANOVA. SUN, sunitinib; PTE, pterostilbene; SUN+PTE, cotreatment with SUN and PTE.
